# MARCH5 inactivation supports mitochondrial function during neurodegenerative stress

**DOI:** 10.3389/fncel.2013.00176

**Published:** 2013-10-10

**Authors:** Lei Fang, Jia Li, Josef Flammer, Albert Neutzner

**Affiliations:** ^1^Department of Biomedicine, University of BaselBasel, Switzerland; ^2^Department of Ophthalmology, The Second Hospital of Jilin UniversityChangchun, China; ^3^Department of Ophthalmology, University of BaselBasel, Switzerland

**Keywords:** MARCH5, mitochondria, Aβ, neurodegeneration, mitochondrial quality control

## Abstract

Neuronal cell death is accompanied by mitochondrial dysfunction with mitochondrial maintenance critical to neuronal survival. The mitochondrial ubiquitin ligase MARCH5 has dual roles in the upkeep of mitochondrial function. MARCH5 is involved in targeted degradation of proteins harmful to mitochondria and impacts mitochondrial morphology upstream of the fission protein Drp1. In a neuronal cell model, dominant-negative MARCH5 prevents mitochondrial fragmentation during neurodegenerative stress induced by the neuron-specific reactive oxygen generator 6-hydroxydopamine, the complex I inhibitor rotenone or Alzheimer’s-related amyloid beta peptide. In addition, preservation of mitochondrial function in terms of membrane potential and lower reactive oxygen generation was observed following inactivation of MARCH5. Our findings connect MARCH5 to neuronal stress responses and further emphasize the link between mitochondrial dynamics and function.

## INTRODUCTION

Mitochondrial dysfunction is at the heart of neurodegeneration (Karbowski and Neutzner, 2012), since neuronal cells are especially dependent on high fidelity mitochondria to meet their extraordinary energy demand. Loss of mitochondrial fidelity due to accumulation of damage is thought to be one of the central mechanisms for the death of neuronal cells associated with virtually all neurodegenerative disorders as well as aging. Damage to mitochondria is caused mainly through reactive reaction intermediates of the mitochondrial electron transport chain (ETC) namely reactive oxygen species (ROS) and reactive nitrogen species (RNS). Main targets of ROS and RNS are mitochondrial proteins as well as mitochondrial DNA but also mitochondrial lipids. Especially damage to proteins of the ETC and to mitochondrial DNA, which codes mainly for ETC components, impacts mitochondrial health as a subpar ETC gives rise to even more reactive intermediates through electron leakage. Various repair, salvage, and maintenance mechanisms are in place to deal with such stresses and to keep mitochondria in a healthy and functional state. On the level of mitochondrial DNA, various repair mechanisms are active, drawing from the large redundancy of mitochondrial DNA with up to 10,000 copies per cell (Bogenhagen and Clayton, 1974). On the protein level, removal of damaged proteins takes place via specialized proteases in the matrix and inner mitochondrial membrane (Rugarli and Langer, 2012) or through the ubiquitin–proteasome system during outer mitochondrial membrane-associated degradation (OMMAD; Neutzner et al., 2007). On the organellar level, the mitochondrial network is maintained through dynamic fission and fusion of mitochondrial tubules essential for adaption of the network to cellular demand (Youle and van der Bliek, 2012). Furthermore, removal of irreparable mitochondrial subunits by mitophagy, a specialized autophagic process, is essential for maintaining organellar fidelity (Youle and Narendra, 2011). Lastly, on the cellular level, irreparable damage to the mitochondrial network causes the induction of apoptosis, thus constituting a complete removal of dysfunctional organelles (Tatsuta and Langer, 2008; Karbowski and Neutzner, 2012). In the case of neuronal tissue with its very limited capacity for regeneration, induction of the cell death program is deleterious and results in the irreparable loss of neuronal function leading ultimately to neurodegenerative disease.

The mitochondrial ubiquitin ligase MARCH5/MITOL (Yonashiro et al., 2006; Karbowski et al., 2007) is involved in maintaining mitochondrial function. MARCH5 was shown to regulate mitochondrial morphology through regulating Drp1 activity (Karbowski et al., 2007), thereby impacting cellular senescence (Park et al., 2010) and modulating neuronal death (Fang et al., 2012). Furthermore, MARCH5 was implicated in the regulation of endoplasmic reticulum (ER)–mitochondrial tethering through ubiquitination of mitofusin Mfn2 (Sugiura et al., 2013). MARCH5 also plays a role in the degradation of mSOD1 associated with amyotrophic lateral sclerosis (Yonashiro et al., 2009), in the disposal of mutated ataxin-3 causative for Machado–Joseph disease (Sugiura et al., 2011) as well as the clearance of *S*-nitrosylated MAP1B-light chain 1 linked to neuronal degeneration (Yonashiro et al., 2012).

Here we found MARCH5 to be involved in the mitochondrial answer to neurodegenerative stress evoked by 6-hydroxydopamine (6-OHDA), a superoxide generating compound selective for neuronal cells, the mitochondrial poison rotenone or Alzheimer’s-related amyloid beta (Aβ). While MARCH5 increased mitochondrial effects of neurodegenerative stress, inactivation of MARCH5 reversed stress-induced fragmentation and strongly ameliorated stress-related mitochondrial dysfunction pointing to an active role of MARCH5 in stress response decisions.

## RESULTS

**FIGURE 1:**
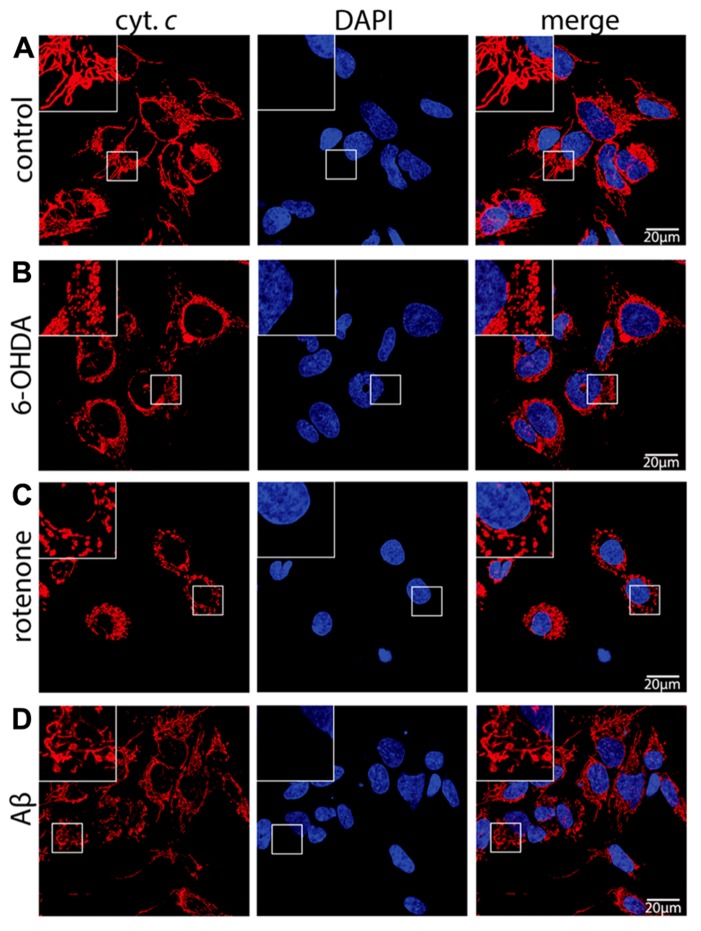
**Neurodegenerative stress causes mitochondrial fragmentation in neuronal cells.** SH-SY5Y cells mock treated (control) **(A)** or exposed for 6 h to 75 μM 6-hydroxydopamine (6-OHDA) **(B)**, for 6 h to 5 μM rotenone (rotenone) **(C)**, or for 24 h to 25 μMAβ peptide (Aβ) **(D)** were fixed and stained using anti-cytochrome *c* antibodies and DAPI to reveal mitochondrial morphology and the nucleus, respectively. Shown are representative pictures of at least three independent experiments.

Here, treatment of SH-SY5Y neuroblastoma cells with the dopaminergic and noradrenergic neuron-specific ROS generator 6-OHDA, the mitochondrial ETC complex I inhibitor rotenone, or the Aβ peptide was employed to study mitochondrial function in a model for neuronal cells under neurodegenerative stress. As revealed by cytochrome *c* staining, following treatment with 6-OHDA (**Figure 1B**), rotenone (**Figure 1C**), or Aβ peptide (**Figure 1D**) the mitochondrial network is considerably fragmented when compared to control cells (**Figure 1A**). The extent of mitochondrial fragmentation was strongest with 6-OHDA, still strong following rotenone and less pronounced after Aβ treatment. These data are consistent with mitochondrial fragmentation in neuronal cells in response to neurodegenerative stress evoked by exogenous ROS, mitochondria-generated ROS as well as mitochondrial dysfunction due to Aβ, respectively.

**FIGURE 2:**
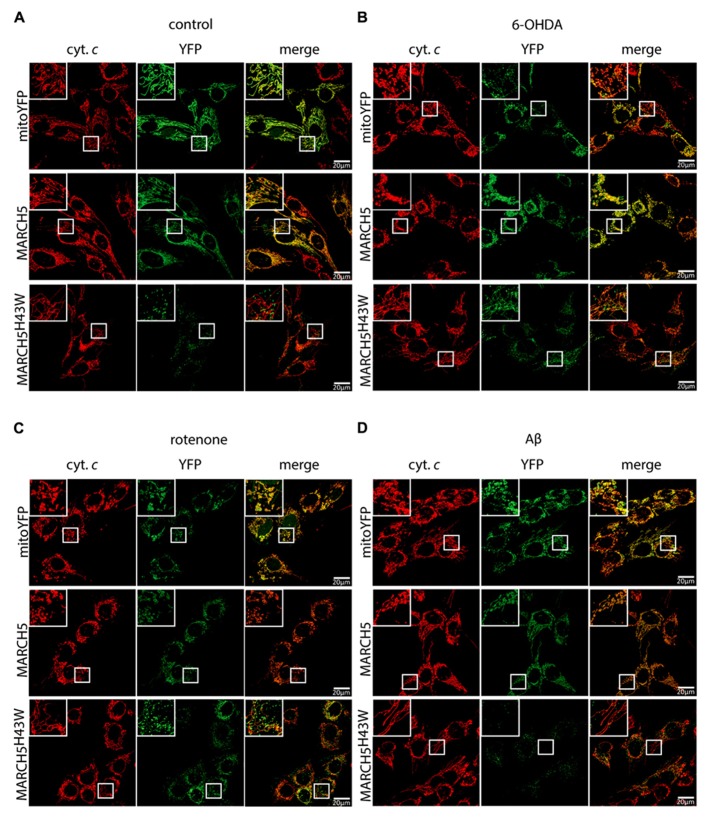
**Inactivation of MARCH5 prevents stress-induced mitochondrial fragmentation.** SH-SY5Y cells selected to express mitochondria-targeted YFP (mitoYFP), MARCH5-YFP, or MARCH5^H43W^-YFP mock treated **(A)** or treated with 75 μM 6-hydroxydopamine for 6 h **(B)**, 5 μM rotenone for 6 h **(C)**, or 25 μM Aβ peptide for 24 h **(D)** were fixed and stained using anti-cytochrome *c* antibodies and imaged using confocal microscopy (cytochrome *c* – red; YFP – green). Shown are representative pictures of at least three independent experiments.

To study the role of MARCH5 in the mitochondrial response to neurodegenerative stress in neuronal cells, wildtype MARCH5, ubiquitin ligase activity negative MARCH5^H43W^, or mitochondria-targeted yellow fluorescent protein (mitoYFP) as control were stably expressed in SH-SY5Y cells. As shown in **Figure 2A** and consistent with previous observations in HeLa cells (Karbowski et al., 2007), expression of MARCH5 in SH-SY5Y cells had no discernible impact on mitochondrial morphology, while expression of dominant-negative MARCH5^H43W^ caused considerable elongation of the mitochondrial network when compared to control cells. In addition and as also reported previously (Karbowski et al., 2007), MARCH5^H43W^ localized to distinct sub-mitochondrial foci in SH-SY5Y cells while wildtype MARCH5 localized to mitochondria in a circumscribing fashion.

To test whether MARCH5 activity is necessary for neurodegenerative stress-induced mitochondrial fragmentation, cells stably expressing MARCH5, MARCH5^H43W^, or mitoYFP were treated with 6-OHDA (**Figure 2B**), rotenone (**Figure 2C**), or Aβ (**Figure 2D**) and mitochondrial morphology was observed following cytochrome *c* staining. While mitochondrial fragmentation was still evident in cells expressing MARCH5 or YFP, expression of MARCH5^H43W^ in SH-SY5Y cells prevented mitochondrial fragmentation evoked by neurodegenerative stress conditions.

**FIGURE 3:**
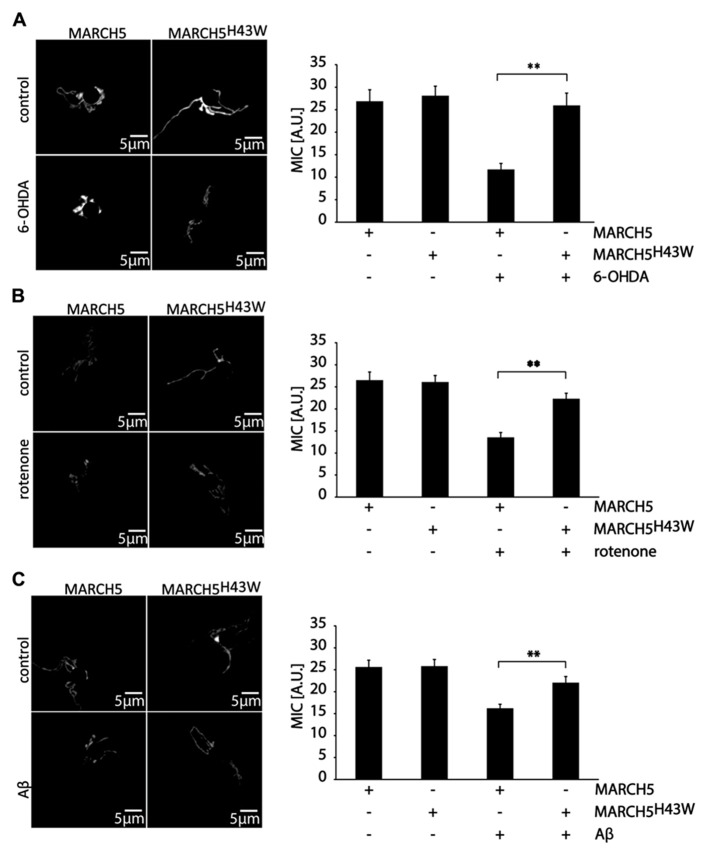
**Inactive MARCH5 supports mitochondrial interconnectivity under neurodegenerative stress conditions.** SH-SY5Y co-transfected with expression plasmids for PA-GFP and either MARCH5 or MARCH5^H43W^ were treated with 75 μM 6-hydroxydopamine for 6 h **(A)**, 5 μM rotenone for 6 h **(B)**, or 25 μMAβ peptide for 24 h **(C)** and mitochondrial interconnectivity was measured following activation of PA-GFP in a small area of the mitochondrial network. Shown is one representative picture and the average of three independent experiments with 15 cells each/condition. Statistical significance was analyzed using Student’s *t*-test with ** marking *p* < 0.01. Error bars represent SEM.

To quantify the impact of MARCH5^H43W^ on preservation of mitochondrial morphology in SH-SY5Y cells following neurodegenerative stress, mitochondrial interconnectivity was measured. To this end, SH-SY5Y cells co-transfected with expression plasmids for MARCH5 or MARCH5^H43W^ and mitochondria-targeted photoactivatable GPF (PA-GFP) were treated with 6-OHDA (**Figure 3A**), rotenone (**Figure 3B**), or Aβ (**Figure 3C**) or mock treated as control and diffusion of PA-GFP was measured following 405 nm laser activation of a small part of the mitochondrial network. The area of PA-GFP of the mitochondrial network covered by activated PA-GFP served as measure for interconnectivity of individual mitochondrial tubules. Treatment of SH-SY5Y cells with 6-OHDA in the presence of MARCH5 expression caused a loss of mitochondrial connectivity compared to mock treated MARCH5 expressing cells, while treatment with 6-OHDA of MARCH5^H43W^ expressing cells had no impact on mitochondrial interconnectivity compared to untreated control cells (**Figure 3A**). Using rotenone as inducer of neurodegenerative stress, blocking MARCH5 function through expression of MARCH5^H43W^ was also able to almost completely suppress loss of mitochondrial interconnectivity (**Figure 3B**). As for treatment of SH-SY5Y cells with Aβ, MARCH5^H43W^ was able to suppress mitochondrial fission compared to MARCH5 expressing cells and preserve connectivity of mitochondrial tubules at levels almost comparable to control cells (**Figure 3C**).

**FIGURE 4:**
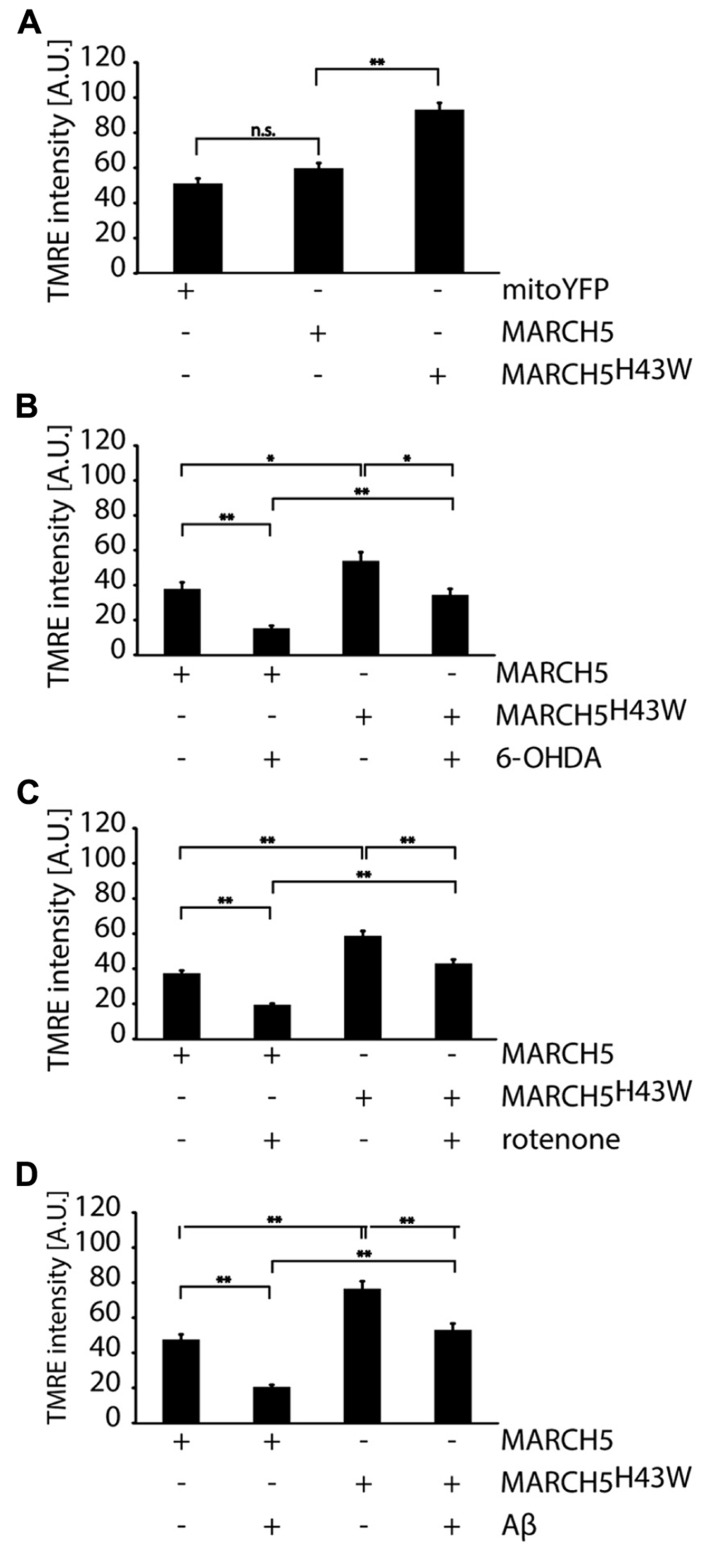
**Mitochondrial membrane potential under neurodegenerative stress conditions is increased following inactivation of MARCH5.** **(A)** SH-SY5Y cells expressing mitoYFP, MARCH5-YFP, or MARCH5^H43W^-YFP were stained with the mitochondrial membrane potential sensitive dye TMRE, images were taken by confocal microscopy andTMRE fluorescence as measure for mitochondrial membrane potential was determined using image analysis. SH-SY5Y cells expressing MARCH5-YFP or MARCH5^H43W^-YFP were treated with 75 μM 6-hydroxydopamine for 6 h **(B)**, 5 μM rotenone for 6 h **(C)**, or 25 μMAβ peptide for 24 h **(D)** and mitochondrial membrane potential was measured as in **(A)**. Shown is the average of three independent experiments with 10 cells each per condition. Statistical significance was analyzed using Student’s *t*-test with * *p*<0.05; n.s. *p*>0.05, not significant and ** marking *p* < 0.01. Error bars represent SEM.

To evaluate whether inactivation of MARCH5 has impact not only on mitochondrial dynamics, but also on mitochondrial core function under neurodegenerative stress conditions, mitochondrial membrane potential in MARCH5 or MARCH5^H43W^ expressing cells following treatment with 6-OHDA, rotenone or Aβ was measured (**Figure 4**) with mock treated cells serving as control. To this end, cells were loaded with the mitochondrial membrane sensitive dye tetramethylrhodamine ethyl ester (TMRE) and single cell analysis of confocal images was performed. While expression of MARCH5 had no effect on mitochondrial membrane potential when compared to mitoYFP expressing control cells, expression of MARCH5^H43W^ caused mitochondrial hyperpolarization (**Figure 4A**). Treatment of MARCH5 expressing cells with 6-OHDA (**Figure 4B**), rotenone (**Figure 4C**), or Aβ (**Figure 4D**) caused significant loss of mitochondrial membrane potential in comparison to untreated MARCH5 expressing cells. Also, treating MARCH5^H43W^ expressing cells with these stressors resulted in a loss of mitochondrial membrane potential compared to untreated MARCH5^H43W^ control cells. However, loss of membrane potential was less pronounced in MARCH5^H43W^ expressing cells between stressed and unstressed conditions compared to wildtype MARCH5 expressing cells. Comparing MARCH5 and MARCH5^H43W^ expressing cells, the membrane potential in stressed MARCH5^H43W^ cells is at levels seen in unstressed MARCH5 expressing cells.

**FIGURE 5:**
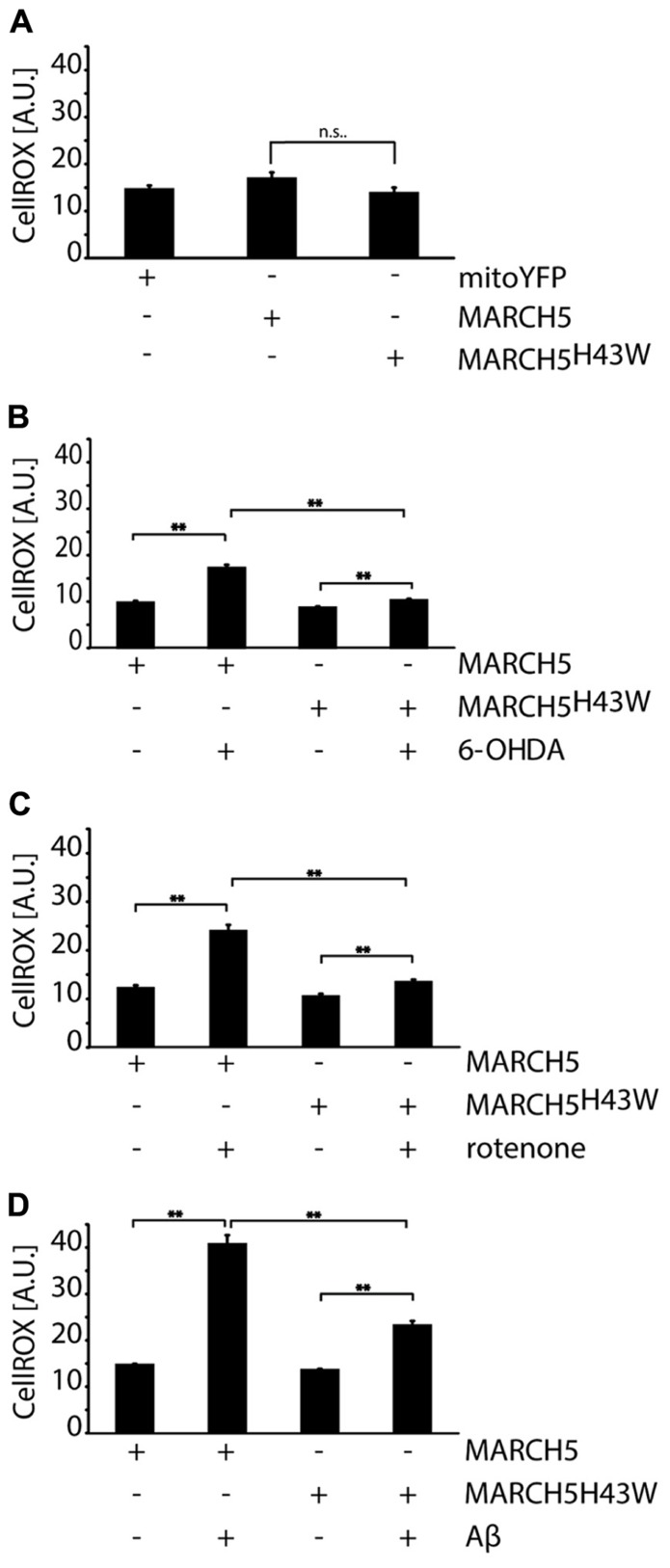
**Inactivation of MARCH5 blunts ROS production under neurodegenerative stress conditions.** **(A)** SH-SY5Y cells expressing mitoYFP, MARCH5-YFP, or MARCH5^H43W^-YFP were treated with the ROS-sensitive dye CellROX and intracellular ROS levels were measured using image analysis of confocal pictures. SH-SY5Y cells expressing MARCH5-YFP or MARCH5^H43W^-YFP were treated with 75 μM 6-hydroxydopamine for 6 h **(B)**, 5 μM rotenone for 6 h **(C)**, or 25 μMAβ peptide for 24 h **(D)** and mitochondrial membrane potential was measured as in **(A)**. Shown is the average of four independent experiments with 10 cells per condition. Statistical significance was analyzed using Student’s *t*-test with ** marking *p* < 0.01 and n.s. marking no significant difference. Error bars represent SEM.

To further gain insight into the mechanisms responsible for MARCH5^H43W^-mediated protection from neurodegenerative stress, cellular levels of ROS were assessed using single cell analysis of CellRox fluorescence. To this end, first, ROS levels were measured in cells expressing MARCH5, MARCH5^H43W^ or mitoYFP and no significant difference between either group was detected (**Figure 5A**). Analysis of MARCH5 or MARCH5^H43W^ expressing cells treated with 6-OHDA revealed an increase in intracellular ROS in MARCH5 cells, while expression of MARCH5^H43W^ prevented this 6-OHDA-mediated spike in ROS almost completely (**Figure 5B**). Similarly, expression of MARCH5^H43W^ was able to blunt ROS production following treatment with rotenone, while ROS levels were elevated about twofold in MARCH5 expressing cells compared to untreated MARCH5 expressing control cells (**Figure 5C**). As for mitochondrial stress evoked by treatment with Aβ, ROS levels in MARCH5^H43W^ expressing cells were about 50% compared to cells producing MARCH5. However, Aβ treatment caused still an increase in ROS even in the presence of inactive MARCH5, albeit to a lower extent compared to control cells (**Figure 5D**).

## MATERIALS AND METHODS

### CELL CULTURE

Human neuroblastoma cell line SH-SY5Y was purchased from DSMZ. SH-SY5Y cells were cultured in high glucose Dulbecco’s modified Eagle’s medium (DMEM), supplemented with 2 mM L-glutamine, 1 mM sodium pyruvate, and 15% fetal bovine serum (Sigma). Cells were incubated in a humidified incubator at 5% CO_2_ and 37°C.

SH-SY5Y cells were transfected using Effectene (Qiagen) according to the manufacturer’s recommendations. To generate stably expressing SH-SY5Y cells, selection was performed using geneticin sulfate (400 μg/ml) for 4 weeks. Degree of transfection was established using flow cytometric analysis (CyAn ADP, Beckman Coulter) and found to be around 80%. SH-SY5Y cells were treated with 6-OHDA (75 μM, 6 h), rotenone (5 μM, 6 h), and Aβ-peptide 25–35 (Sigma A4559, 25 μM, 24 h).

### MICROSCOPY

For immunocytochemistry, cells were seeded in six-well plates onto glass slides at 1 × 10^4^ cells/well in 2 ml culture medium. Samples were fixed using methanol-free electron microscopy grade 4% paraformaldehyde in phosphate buffered saline (PBS) for 15 min at room temperature (RT), permeabilized for 15 min at RT using 0.15% Trixon X-100 in PBS and blocked for 1 h in 10% bovine serum albumin (BSA) in PBS. To visualize mitochondria, samples were then incubated with mouse anti-cytochrome *c* antibody (BD Biosciences 556432, 1:1000) overnight at 4°C and Alexa546-conjugated anti-mouse antibodies (Invitrogen A11003, 1:500) for 1 h at RT. Samples were mounted in mounting medium (Vectashield H1000) and observed using a confocal microscope (Zeiss LSM Meta710, 63×/1.4 objective). For life cell imaging, cells were seeded onto chambered coverglass (Nunc Lab-Tek, 154461) at a density of 5 × 10^3^/well in 1 ml culture media. Measurement of mitochondrial interconnectivity was performed as described before (Fang et al., 2012). In short, SH-SY5Y cells were transfected with an expression construct for PA-GFP and mitochondrial network area following photoconversion of PA-GFP employing a 405-nm laser was measured. To measure mitochondrial membrane potential, cells were stained with 100 nM TMRE (Invitrogen, T-669) in media for 20 min at 37°C and washed three times with media. Imaging was performed on a LSM710 confocal microscope (Zeiss) equipped with a live cell imaging chamber. Z-stacks (five images, 1 μm distance) were acquired and image analysis was performed using Imaris v7.0 software (Bitplane Scientific Software). Data are expressed as mean signal intensity of 30 randomly selected cells per treatment group (three independent experiments, 10 cells each). To measure cellular ROS, cells were stained using 5 μM CellROX Deep Red Reagent (Invitrogen, C10422) for 30 min at 37°C, washed three times with PBS and fixed using 4% paraformaldehyde before imaging. Z-stacks (five images at 1 μm intervals) were acquired and analyzed using Imaris 7.0. Data are expressed as mean signal intensity of cells (four independent experiments, 10 cells each/group each).

### STATISTICAL ANALYSIS

Statistical analysis was performed using unpaired, two-tailed Student’s *t*-test as implemented in Microsoft Excel. A *p*-value of <0.05 or smaller was considered statistically significant and is marked with *, while *p*-values of <0.01 are marked with **. Error bars represent the standard error of the mean (SEM).

## DISCUSSION

While MARCH5 is involved in the removal of proteins associated with specific neurodegenerative disorders such as ataxin-3 in Machado–Joseph disease or mSOD1 in amyotrophic lateral sclerosis likely supporting mitochondrial function, MARCH5 activity during general mitochondrial oxidative stress does not seem to confer a protective effect. Mitochondrial fragmentation in response to oxidative insults evoked by external ROS generators such as 6-OHDA or internal ROS generators such as rotenone or Aβ was greatly diminished in cells expressing MARCH5^H43W^, while wildtype MARCH5 did not prevent the remodeling of the mitochondrial network in response to stress. Thus it is conceivable that during neurodegenerative stress, removal of damaged proteins from mitochondria through MARCH5 seems not to be as essential as one might expect from an ubiquitin ligase involved in mitochondrial protein quality control. Rather, the function of MARCH5 as mitochondrial morphogen modulating Drp1 activity might be important in this context. And indeed we previously showed that inactivation of MARCH5 protects neuronal cells from stress-induced cell death (Fang et al., 2012) likely through the inhibition of Drp1-dependent mitochondrial fragmentation in accordance with previous observations where inhibition of Drp1 activity strongly delayed cell death (Frank et al., 2001). Thus, the function of MARCH5 in regulating mitochondrial morphology might also be the main factor in the here observed positive effect on mitochondrial fidelity upon expression of dominant-negative MARCH5^H43W^. Fragmentation of the mitochondrial network is a response to potentially lethal stress conditions such as increased oxidative stress or loss of membrane potential. As mitochondrial fragmentation is an integral part of the apoptotic program with forced fragmentation sensitizing cells to apoptotic stimuli (Lee et al., 2004), shortening mitochondria does not seem to have a protective effect on cells but might rather be seen as preparation for starting the cell death program although mitochondrial fission *per se* is not an apoptotic stimulus (Youle and van der Bliek, 2012). In contrast, elongation of mitochondria seems to be protective as evidenced by de-sensitization to apoptotic stimuli following increased mitochondrial fusion (Neuspiel et al., 2005). Also during stress-induced mitochondrial hyperfusion (SIMH), enhanced fusion and therefore highly interconnected mitochondrial tubules prove to increase resistance against certain stresses (Tondera et al., 2009). While most experimental stress conditions induce mitochondrial fragmentation, stress at levels well below the apoptotic threshold induces mitochondrial elongation. The SIMH-associated adaption of mitochondrial morphology is brought about in an Mfn1- and OPA1-dependent but Mfn2-independent manner and is likely not achieved by forced mitochondrial elongation due to inhibition of Drp1 function (Tondera et al., 2009). The increased interconnectivity during SIMH conditions mitochondria against further stress potentially by boosting their ATP production likely via increased availability of substrates and ETC intermediates in fused mitochondrial reticulum with its extended matrix space. Whether inactivation of MARCH5 induces SIMH is unclear as mitochondrial elongation in the absence of external stress is evident in MARCH5^H43W^ expressing cells, however, the observed increase of mitochondrial membrane potential in unstressed cells and the blunting of stress-induced ROS production point in this direction. This leaves the question, which additional pathways besides inhibition of Drp1-mediated fission might be influenced by MARCH5^H43W^ as simply inhibiting Drp1 does not seem to evoke SIMH (Tondera et al., 2009). Based on our results, one might speculate that MARCH5 as upstream regulator of Drp1 is important during cellular stress responses and might modulate the activity of other targets besides Drp1. The notion of MARCH5 regulating other such targets is supported by our observation that expression of wildtype MARCH5 did not preserve mitochondrial function under neurodegenerative stress conditions although no effect on mitochondrial interconnectivity was observed (Karbowski et al., 2007). Importantly, expression of MARCH5 in the absence of stress conditions did not impact mitochondrial membrane potential or ROS production further hinting to a role for MARCH5 during mitochondrial stress. Thus, based on our observations following dominant-negative MARCH5^H43W^ expression and the effects of wildtype MARCH5, it seems conceivable that MARCH5 is involved in the decision for stress-induced fragmentation versus protective mitochondrial elongation. Taken together, our data further support a role of MARCH5 in the modulation of Drp1 activity during mitochondrial fission and implicate MARCH5 in mitochondrial stress response pathways. As the mitochondrial stress response is pathophysiologically significant from diabetes to cardiovascular disease to neurodegeneration, blockage of MARCH5 might be an interesting therapeutic strategy.

## Conflict of Interest Statement

The authors declare that the research was conducted in the absence of any commercial or financial relationships that could be construed as a potential conflict of interest.

## AUTHOR CONTRIBUTIONS

Lei Fang, Josef Flammer, and Albert Neutzner conceived experimental design; Lei Fang and Jia Li performed experiments; Lei Fang and Albert Neutzner wrote the article.
